# A unique homozygous WRAP53 Arg298Trp mutation underlies dyskeratosis congenita in a Chinese Han family

**DOI:** 10.1186/s12881-018-0549-1

**Published:** 2018-03-07

**Authors:** Yingqi Shao, Sizhou Feng, Jinbo Huang, Jiali Huo, Yahong You, Yizhou Zheng

**Affiliations:** State Key Laboratory of Experimental Hematology, Institute of Hematology & Blood Diseases Hospital, Chinese Academy of Medical Science & Peking Union Medical College, 288 Nanjing Road, Tianjin, 300020 People’s Republic of China

**Keywords:** WRAP53, Dyskeratosis congenita, Homozygous

## Abstract

**Background:**

Dyskeratosis congenita (DC) is an inherited telomeropathy characterized by mucocutaneous dysplasia, bone marrow failure, cancer predisposition, and other somatic abnormalities. Cells from patients with DC exhibit short telomere. The genetic basis of the majority of DC cases remains unknown.

**Methods:**

A 2 generational Chinese Han family with DC was studied using targeted capture and next-generation sequencing to identify the underlying DC-related mutations.

**Results:**

In this study, we identified a unique homozygous WD repeat containing antisense to TP53 (WRAP53) Arg298Trp mutation in the proband with DC and heterozygous WRAP53 Arg298Trp mutations in his asymptomatic, consanguineous parents and his sister, indicating an autosomal recessive inheritance mode. The proband with the homozygous WRAP53 Arg298Trp mutation had short telomere, classic clinical symptoms, and no response to danazol, glucocorticoid or cyclosporin A.

**Conclusions:**

Thus, we reported for the first time that a unique homozygous WRAP53 mutation site underlies the development of DC.

## Background

Dyskeratosis congenita (DC) is an inherited telomeropathy characterized by ectodermal dysplasia, bone marrow failure (BMF), cancer predisposition and other somatic abnormalities. The typical triad of nail dysplasia, skin pigmentation and oral leukoplakia has clinical diagnostic value; DC is also associated with the risk of developing trilineage BMF, pulmonary fibrosis, liver fibrosis and cirrhosis, malignancies, developmental delays, and stenosis of the esophagus, urethra, or lacrimal ducts [1–4]. However, the clinical symptoms of DC are highly heterogeneous, making it difficult to diagnose DC with confidence based on clinical features alone.

A significant advance in the understanding of the pathogenic mechanism of DC came from the discovery that dyskerin, a protein encoded by DKC1, which was the first gene identified in X-linked DC patients [5], was associated with telomerase and that dyskerin mutations caused a reduction in telomerase RNA component (TERC) and telomere length [6]. After these findings, studies over the next 10 years showed that DC was fundamentally a telomere disease in which patients showed premature loss of telomere repeats, and the loss of functional telomere structure leading to early cell death/senescence and a wide variety of clinical features [6–8]. To date, there are eleven reported DC-associated genes (DKC1, TERT, TERC, TINF2, WRAP53, NOP10, NHP2, CTC1, RTEL1, PARN and TPP1) [5, 9–16] that are responsible for the function or maintenance of telomeres [17, 18]. The inheritance of DC is genetically heterogeneous, including autosomal dominant inheritance (TERT, TERC, TINF2, TPP1 or RTEL1), autosomal recessive inheritance (TERT, WRAP53, NOP10, NHP2, CTC1, PARN or RTEL1), and X-linked inheritance (DKC1) [19, 20].

In 2011, compound heterozygous WD repeat containing antisense to TP53 (WRAP53) mutations were identified in 2 unrelated DC patients with autosomal recessive inheritance [21]; that study was the only report thus far of WRAP53 mutations in DC. The two unrelated probands presented the classical triad of skin pigmentation, oral leukoplakia, and nail dysplasia, and the peripheral lymphocytes of the probands had shortening telomere lengths. Parents and siblings of the probands harbored heterozygous WRAP53 mutations and had normal telomere lengths, showing that it was an autosomal recessive inheritance [21]. WRAP53 is associated with telomerase and is required for its delivery to the telomeres during the S phase [22]. WRAP53 is associated with TERC, TERT, dyskerin, and small Cajal body RNAs [22] and mediates the localization of H/ACA class RNAs to Cajal bodies through the recognition of a CAB box [22, 23]. Knockdown of WRAP53 causes the loss of TERC and dyskerin from the Cajal bodies, mislocalization of TERC in the nucleolus, and progressive telomere shortening [21, 22].

In this work, we identified a unique homozygous DC-associated WARP53 Arg298Trp mutation with an autosomal recessive inheritance mode in a DC patient who was an offspring of a consanguineous marriage.

## Methods

### Patients

A patient with consanguineous parents presented with pancytopenia in 2017 and was referred to the Institute of Hematology & Blood Diseases Hospital, Chinese Academy of Medical Science (CAMS) & Peking Union Medical College (PUMC), Tianjin, China. He was diagnosed with DC. We invited the entire family for testing for mutations in WRAP53, and three additional family members consented to participate in this study. The study was approved by the Ethics Committees of the Institute of Hematology, CAMS and PUMC (ethics number: KT2014005-EC-1).

### Targeted capture and next-generation sequencing (NGS)

Genomic DNA was extracted from peripheral blood samples following standard procedures using genomic DNA kits (QIAamp DNA Blood MiniKit, Qiagen), and the extracted DNA was sheared into fragments ranging from 200 to 300 bp by an ultrasonoscope (Covaris S2, Massachusetts, USA). An NGS assay was designed to cover all exons, splice sites, and the flanking intron sequences of 9 DC-associated genes (DKC1, TERC, TERT, NHP2, NOP10, CTC1, RETL1, WRAP53, and TINF2). Following the standard procedures, an Illumina TruSeq custom amplicon kit was used for library preparation, and a Nimblegen Sequence Capture array was used for sequence capture. Illumina Pipeline software (version 1.3.4) was used to analyze the primary sequencing data. SOAPsnp software and Sam tools pileup software were used to analyze the gene mutations.

### Confirmation of candidate mutation by sanger sequencing

To confirm the WRAP53 Arg298Trp mutation identified by NGS, the corresponding WRAP53 gene region surrounding the mutation was amplified by PCR and sequenced by Sanger sequencing. PCR amplifications were performed using an Applied Biosystems® GeneAmp® PCR System 9700. The sequence was loaded on an ABI 3730XL automat (Applied Biosystems). The sequence data files were analyzed using both Sequencing Analysis 5.2 and Variant Reporter v 1.1 software (Applied Biosystems). The primers used to validate the WRAP53 Arg298Trp mutation were as follows:

- Forward primer: ATCTGGGACGCATTCACTGG.
- Reverse primer: TGAGCCTGTTTGCTCCTCTG.
- Product length: 456 bp.

### Telomere length analysis

Telomere length measurements of peripheral blood lymphocytes were performed by flow cytometry with fluorescence in situ hybridization analysis (flow-FISH) [24] by a testing center (NanTian Biotech), which provided references to the telomere lengths of age-matched healthy controls.

### Colony assay

Mononuclear cells (MNCs) were sorted from the patient’s BM sample and were seeded in 24-well plates at a density of 1 × 10^5^ cells/well in a medium containing methylcellulose medium, 50 ng/mL FL, 50 ng/mL SCF, 20 ng/mL TPO and 20 ng/mL IL-3. The culture was incubated at 37 °C in a humidified atmosphere that contained 5% CO_2_. The colonies were scored at day 10.

## Results

**Fig. 1:**
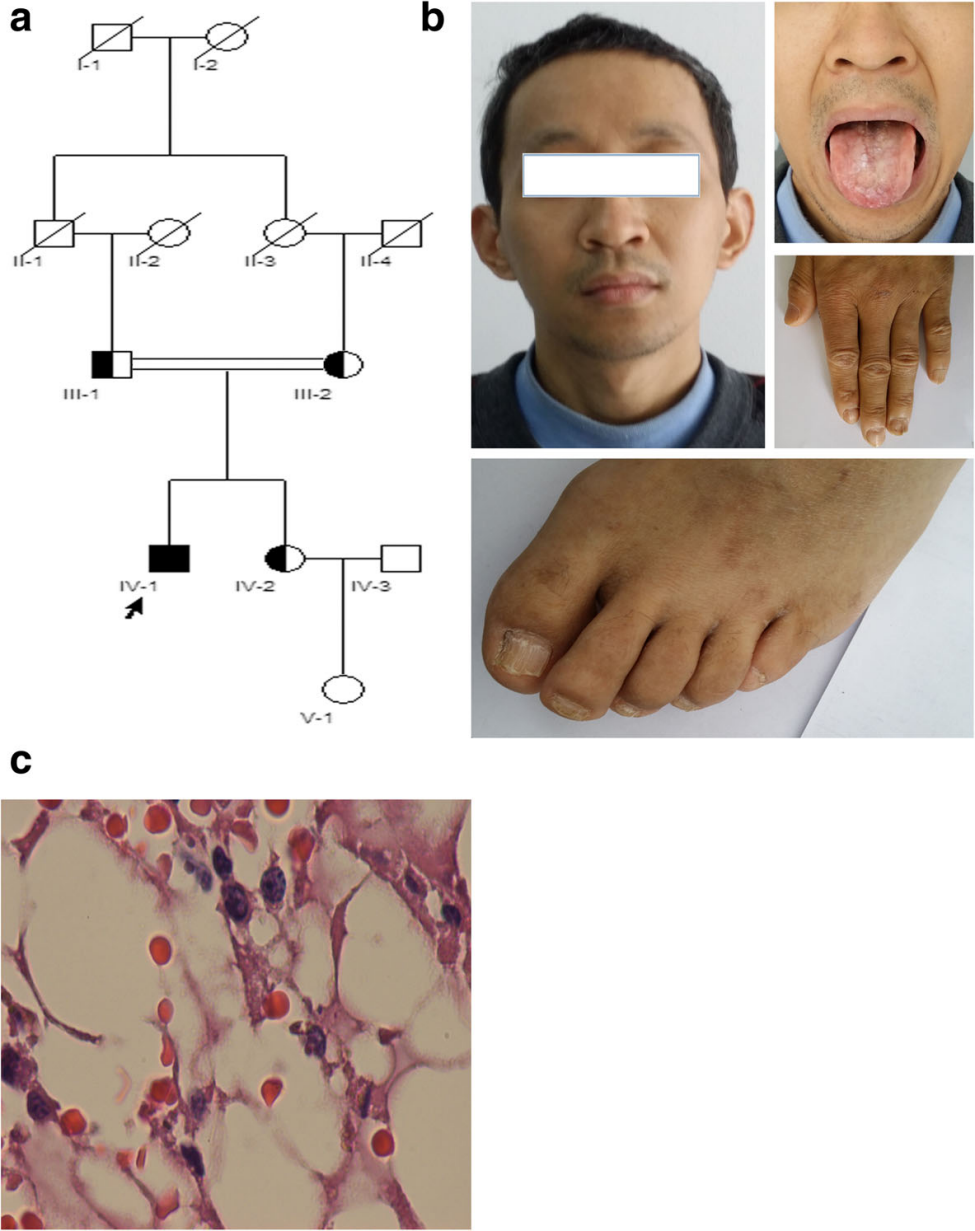
**a** Pedigree of a Chinese family affected with DC. The arrow indicates the proband with typical clinical symptoms. His heterozygous, consanguineous parents and sister had no clinical symptoms. **b** The affected proband with DC showed the typical signs of nail dysplasia and oral leukoplakia. **c** Bone marrow biopsy of the proband revealed severe hypocellularity (HE stain, × 400)

In this study, four individuals from a 2 generation family were enrolled for investigation of an underlying DC-related gene mutation. The recruited individuals consisted of the proband (IV-1, 30 years), his asymptomatic sister (IV-2, 34 years), his asymptomatic consanguineous father (III-1, 58 years) and mother (III-2, 55 years). Pedigree analysis showed an autosomal recessive inheritance mode (Fig. 1a).

### Clinical findings

The proband (IV-1) was a 30-year-old Han Chinese male who was born in a rural village in Sichuan Province, China, and who was the second child of consanguineous parents with no family history of BMF. Pancytopenia (WBC = 2.73 × 10^9^/L, Hb = 94 g/L and PLT = 12 × 10^9^/L) and moderate splenomegaly were revealed by regular medical examination at age 27. Further examination showed hypocellularity of his iliac BM, and liver cirrhosis and portal hypertension were diagnosed at age 28 at West China Hospital, Sichuan University in Sichuan Province, Chengdu, China. Although no definite diagnosis was made during the following 2 years, the symptoms became worse, and the patient became transfusion-dependent with time. He was referred to our center to seek further medical help. Physical examination revealed his short stature, leukoplakia on his buccal mucous membrane, dystrophic distal ends of the fingernail and toenails, and skin with spotted pigmentation in the posterior chest and neck (Fig. 1b). Complete blood counts revealed a Hb level of 48 g/L, an MCV of 91.7 fl, a WBC of 0.4 × 10^9^/L, a PLT of 6 × 10^9^/L, and a reticulocyte count of 0.0116 × 10^12^/L. The liver function test showed normal functioning (ALT = 33.70 U/L, AST = 30.10 U/L, TBIL = 14.70 μmol/L). Abdominal ultrasonography identified liver cirrhosis, marked splenomegaly and normal kidneys. BM aspiration and biopsy confirmed the hypocellularity of the BM (Fig. 1c). A cytogenetic study showed a normal karyotype (46, XY [20]). There was no increase in the chromosomal breakage as measured by single cell gel electrophoresis (SCGE) and mitomycin C (MMC) tests. Pulmonary function was normal. The proband’s sibling (IV-2) and parents (III-1 and III-2) had no mucocutaneous symptoms, normal complete blood counts, and no hepatosplenomegaly but were not further examined by BM aspiration, biopsies or pulmonary function tests.

### Genetic findings

**Fig. 2:**
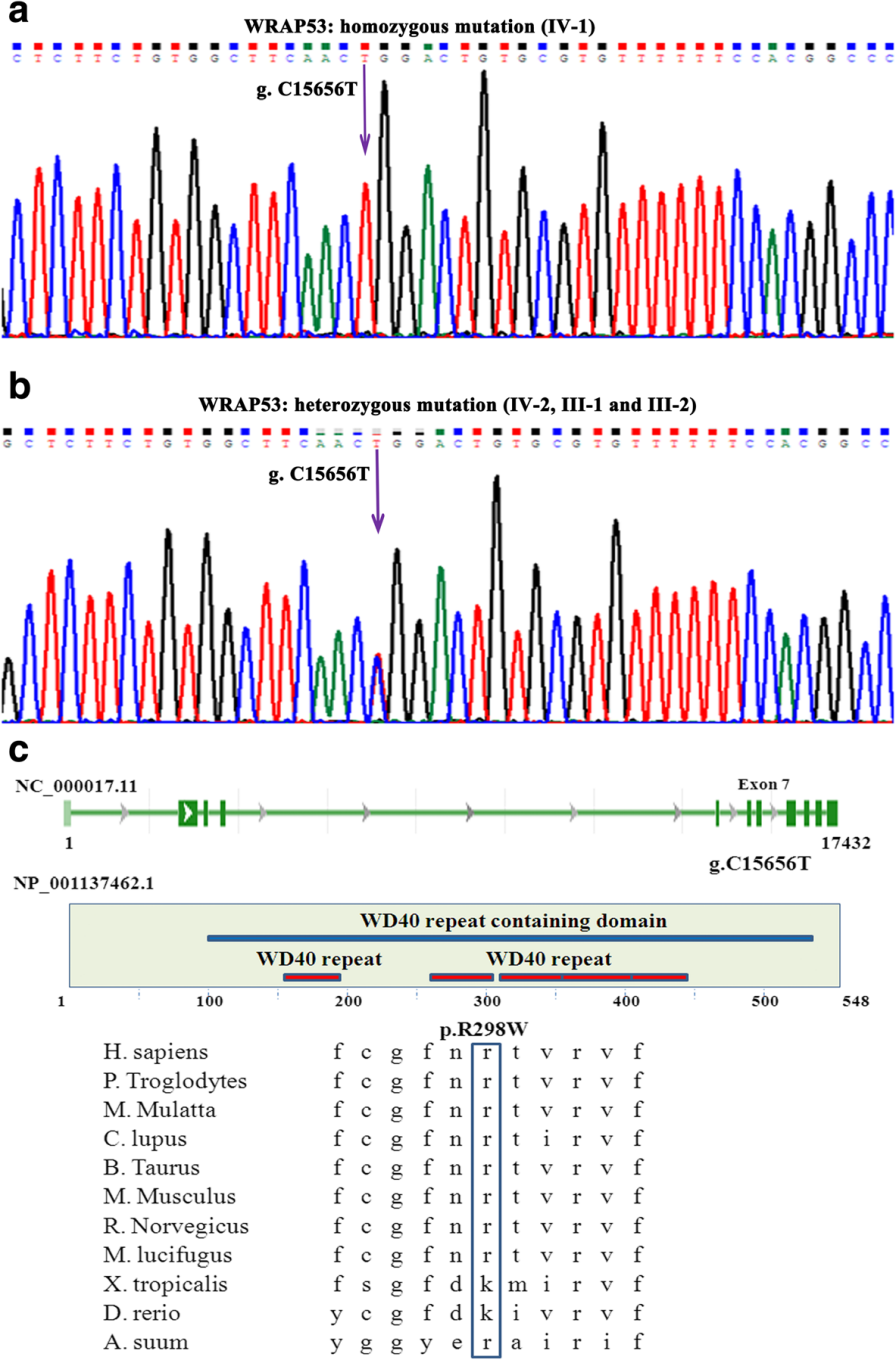
**a** Direct sequencing of the WRAP53 gene identified a homozygous mutation (c.C892T, p.R298W) in the proband with DC. **b** Direct sequencing of the WRAP53 gene revealed that the proband’s parents and sister harbored the heterozygous mutation. **c** (Top) Location of the mutated nucleotides in the scheme of the WRAP53 nucleotide structure. (Middle) Location of the mutated amino acid in the scheme of the WRAP53 protein structure. (Bottom) Multi-species sequence comparison of WRAP53 shows that R298 is evolutionarily conserved

In view of the classic DC-related mucocutaneous symptoms of the proband (IV-1), genetic testing for known DC-related genes (DKC1, TERC, TERT, NHP2, NOP10, CTC1, RETL1, WRAP53, and TINF2) was performed, and the mutation analysis revealed that the proband (IV-1) harbored a unique homozygous missense mutation for a C to T transition in exon 7 of the WRAP53 gene, causing an arginine (R) to tryptophan (W) substitution at amino acid position 298 (NM_001143990: c.C892T, p.R298W) (Fig. 2a and c). The tests for mutations of DKC1, TERC, TERT, NHP2, NOP10, CTC1, RETL1, and TINF2 were negative. The asymptomatic sister (IV-2), father (III-1), and mother (III-2) had heterozygous WRAP53 Arg298Trp mutations (Fig. 2b and c), implying that DC with the WRAP53 mutation has an autosomal recessive inheritance mode. The position of the WRAP53 Arg298Trp mutation is in the second WD40 domain of the WRAP53 protein, a conserved domain found in several eukaryotic proteins that cover a wide variety of functions including adaptor/regulatory modules in signal transduction, premRNA processing and cytoskeleton assembly (Fig. 2c).

We used three bioinformatics tools to analyze the impact of the Arg298Trp mutation on the structure and function of WRAP53. The possible impact of the Arg298Trp mutation on the structure and function of WRAP53 was predicted by the PolyPhen-2 online server (http://genetics.bwh.harvard.edu/pph2/), and the mutation was predicted to be damaging, with a score of 1.000 (sensitivity: 0.00; specificity: 1.00). The change in protein stability with the Arg298Trp mutation was computed by the DUET web server (http://structure.bioc.cam.ac.uk/duet), and the mutation was predicted to be destabilizing. The MutationTaster online server (http://www.mutationtaster.org/) was used to predict the association between the Arg298Trp mutation and disease, and the mutation was predicted to be disease causing.

### The short telomere in the DC patient with the WRAP53 Arg298Trp mutation

**Fig. 3:**
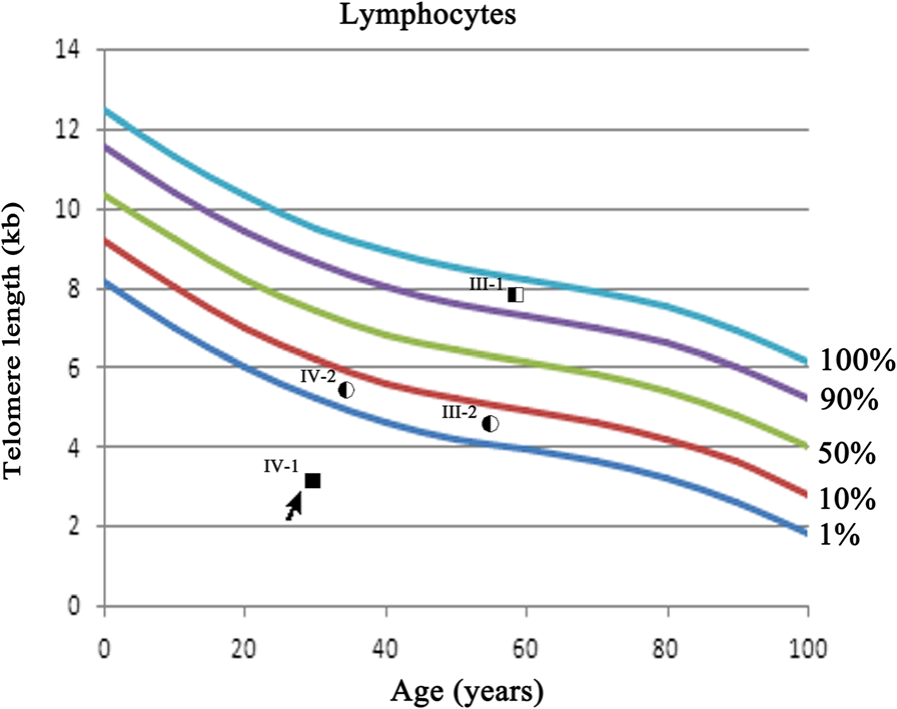
Measurement of telomere length by flow-FISH in the peripheral blood lymphocytes. Absolute telomere lengths in kb of the lymphocytes of the proband (IV-1), his sibling (III-2) and his parents (III-1 and III-2) are shown in the context of the age-dependent percentiles

The telomere length in the peripheral blood lymphocytes from the proband (IV-1) corresponded to less than the 1st percentile of age-matched controls (Fig. 3). Compared to the age-matched controls, the asymptomatic sibling (III-2) and mother (III-2) also displayed decreased telomere lengths, although not below the 1st percentile; however, the asymptomatic father (III-1) had normal telomere lengths (Fig. 3).

### MNCs from the DC patient’s BM showed hypoproliferation ex vivo

BM puncture and biopsy of the proband (IV-1) showed marked hypocellularity (< 10%), but no evidence of myelodysplasia or myelofibrosis. Subsequently, a colony assay was performed; 1 × 10^5^ sorted BMMNCs from the proband (IV-1) were seeded into methylcellulose cultures, and the colonies were counted at day 10. The colonies of CFU-E, BFU-E, CFU-GM and CFU-mix were 30 (normal reference range: 68–93), 10 (normal reference range: 25–37), 14 (normal reference range: 14–29) and 0 (normal reference range: 0–1), respectively. Compared with the normal control, the colony formation of BMMNCs from the proband (IV-1) was significantly lower.

### The DC patient with the WRAP53 Arg298Trp mutation did not respond to danazol, glucocorticoid or cyclosporin A (CsA)

The proband accepted treatment with danazol, glucocorticoid and/or CsA during the 3 years following the initial presentation (although no definite diagnosis had been made then), but had no response; his symptoms became worse, and he became transfusion dependent over time. In addition, he progressed to liver cirrhosis and portal hypertension, and his spleen also enlarged with time. Splenomegaly was revealed during his visit to our center, which may have further aggravated pancytopenia by the peripheral destruction of blood cell components by hypersplenism.

## Discussion

The clinical presentation of DC is highly variable, and little is known about the physiopathological mechanisms underlying that variability. The clinical diagnosis of DC may be challenging due to different ages of onset, varied clinical features and genetic heterogeneity. In this study, we identified a DC patient with a unique homozygous WRAP53 mutation with consanguineous parents. The onset age of the patient with the homozygous WRAP53 mutation was 27 years (which was relatively late), and he presented classic symptoms, BMF and liver cirrhosis.

Compound heterozygous WRAP53 mutations were identified in 2 unrelated patients with DC [21] and, until now, that was the only report about WRAP53 mutations in DC patients. In this study, we revealed a homozygous WRAP53 mutation in a DC patient with consanguineous parents. WRAP53 is a scaffold protein that acts as a platform for the assembly of large protein and RNA complexes to facilitate their interactions. WRAP53 mediates the localization of the specific H/ACA class RNAs to Cajal bodies through the recognition of a CAB box [22, 23]. WRAP53 joins in the activation of telomerase and is associated with the delivery of telomerase to the telomeres during the S phase [22]. WRAP53 is involved in DNA repair, the maintenance of Cajal bodies, and telomere elongation [22, 25]. Knockdown of WRAP53 causes mislocalization of TERC to the nucleolus, the loss of TERC and dyskerin from Cajal bodies, and progressive telomere shortening over time [21, 22]. Mutations in WRAP53 cause the misdirection of telomerase RNA to the nucleoli, inhibit the localization of the telomeres to Cajal bodies, and prevent telomerase from elongating the telomeres, which results in the shortening of the telomeres. WRAP53 contains five WD40 repeats that appear to be critical to its function. WD40 repeats are a protein structure, and they mediate a variety of cellular processes by serving as a scaffold for multiple interactions between molecules via flexible loops. In our case, the mutated region of WRAP53 was in the second WD40 domain. Disease-associated mutations in WRAP53 are all located in that highly conserved residue and are predicted to change the function of the protein [26]. Arg298 is in the very highly conserved residue of the WRAP53 protein. We used three bioinformatics tools to analyze the impact of the Arg298Trp mutation on the structure and function of WRAP53.The mutation was predicted to be damaging, with a score of 1.000, protein destabilizing and disease causing. A previous study showed that DC-associated WRAP53 mutations contribute to a marked reduction in endogenous WRAP53 levels and prevent it from localizing to Cajal bodies [21]. The proband’s parents and sister had a heterozygous WRAP53 mutation and had no clinical symptoms, indicating that WRAP53 mutation has an autosomal recessive inheritance mode, which agrees with the previous report [21].

DC patients have shorter telomeres than healthy individuals, but there is substantial variation in different patients [27]. Flow-FISH has been widely used to detect telomere length in the leukocytes of DC patients, and telomere lengths in lymphocytes less than the first percentile has been shown to be a sensitive and specific test to differentiate DC patients from healthy individuals [28]. In this study, we showed that the telomere length in peripheral blood lymphocytes from the proband was significantly shorter, corresponding to less than the 1st percentile of age-matched controls, which implies that a telomere length less than the 1st percentile could be used as a significant marker for DC in the clinical setting. The proband’s asymptomatic sister, father, and mother had heterozygous WRAP53 Arg298Trp mutations; the telomere lengths in the peripheral blood lymphocytes from the sibling and the mother were less than the 10th percentile of age-matched controls, and the father had a normal telomere length. In a previous report, parents with heterozygous WRAP53 mutations also had normal telomere lengths [21], suggesting that heterozygous WRAP53 mutation may not be a critical factor affecting telomere length. In fact, telomere inheritance, chronic inflammatory diseases, confounding environmental factors and lifestyle can all affect the mean PBMC telomere length of an individual.

DC is a multiorgan disorder, and BMF and liver cirrhosis are the severe complications. The DC patient with the homozygous WRAP53 mutation had marked hypocellularity of the BM, liver cirrhosis, portal hypertension, marked splenomegaly, and had no response to danazol, glucocorticoid and/or CsA. BMF in DC patients does not respond to immunosuppressive therapy, including CsA and antithymocyte globulin [29]. In total, 50%–70% of DC patients respond to androgens, achieving red blood cell and/or platelet transfusion independence [30–32]. The DC patient with the homozygous WRAP53 mutation did not respond to danazol, and the liver cirrhosis, portal hypertension and marked splenomegaly may be some of the factors influencing that lack of response. In addition, the patient had esophageal varices and seriously decreased platelet levels (often could not receive platelet transfusion in a timely manner because of his poor economic situation), and he often suffered from gastrointestinal hemorrhage, which may have further aggravated the symptoms and affected the absorption of the drugs.

## Conclusions

In conclusion, our study is the first to report a unique homozygous WRAP53 mutation site in a DC patient. The DC patient harboring the homozygous WRAP53 Arg298Trp mutation had shortened telomeres, classic clinical symptoms, BMF, liver cirrhosis and poor response to therapy.

## Abbreviations

BMF: Bone marrow failure
CsA: Cyclosporin A
DC: Dyskeratosis congenita
flow-FISH: Fluorescence in situ hybridization analysis
MMC: Mitomycin C
MNCs: Mononuclear cells
SCGE: Single cell gel electrophoresis
TERC: Telomerase RNA component
WRAP53: WD repeat containing antisense to TP53
